# Prospective healthcare-associated links in transmission of nontuberculous mycobacteria among people with cystic fibrosis (pHALT NTM) study: Rationale and study design

**DOI:** 10.1371/journal.pone.0291910

**Published:** 2023-12-20

**Authors:** Jane E. Gross, Silvia Caceres, Katie Poch, L. Elaine Epperson, Nabeeh A. Hasan, Fan Jia, Vinicius Calado Nogueira de Moura, Matthew Strand, Ettie M. Lipner, Jennifer R. Honda, Michael Strong, Rebecca M. Davidson, Charles L. Daley, Jerry A. Nick

**Affiliations:** 1 Department of Pediatrics, National Jewish Health, Denver, CO, United States of America; 2 Department of Medicine, National Jewish Health, Denver, CO, United States of America; 3 Center for Genes, Environment and Health, National Jewish Health, Denver, CO, United States of America; 4 Division of Biostatistics, National Jewish Health, Denver, CO, United States of America; 5 Epidemiology and Population Studies Unit, National Institute of Allergy and Infectious Diseases, NIH, Bethesda, MD, United States of America; 6 Department of Medicine, University of Colorado School of Medicine, Aurora, CO, United States of America; Shandong Public Health Clinical Center: Shandong Provincial Chest Hospital, CHINA

## Abstract

**Background:**

Healthcare-associated acquisition and transmission of nontuberculous mycobacteria (NTM) among people with cystic fibrosis (pwCF) has been described, and remains a concern for both patients and providers. This report describes the design of a prospective observational study utilizing the standardized epidemiologic investigation toolkit for healthcare-associated links in transmission of NTM among pwCF.

**Methods:**

This is a parallel multi-site study of pwCF who have infections with respiratory NTM isolates and receive healthcare within a common CF Care Center. Participants have a history of one or more NTM positive airway cultures and have been identified as having NTM infections suggestive of a possible outbreak within a single Center, based on NTM isolate genomic analysis. Participants are enrolled in the study over a 3-year period. Primary endpoints are identification of shared healthcare-associated source(s) among pwCF in a Center, identification of healthcare environmental dust and water biofilm NTM isolates that are genetically highly-related to respiratory isolates, and identification of common home of residence watersheds among pwCF infected with clustered isolates. Secondary endpoints include characterization of healthcare-associated transmission and/or acquisition modes and settings as well as description of incidence and prevalence of healthcare-associated environmental NTM species/subspecies by geographical region.

**Discussion:**

We hypothesize that genetically highly-related isolates of NTM among pwCF cared for at the same Center may arise from healthcare sources including patient-to-patient transmission and/or acquisition from health-care environmental dust and/or water biofilms. This study design utilizes a published, standardized, evidence-based epidemiologic toolkit to facilitate confidential, independent healthcare-associated NTM outbreak investigations within CF Care Centers. This study will facilitate real-time, rapid detection and mitigation of healthcare-associated NTM outbreaks to reduce NTM risk, inform infection prevention and control guidelines, and characterize the prevalence and origin of NTM outbreaks from healthcare-associated patient-to-patient transmission and/or environmental acquisition. This study will systematically characterize human disease causing NTM isolates from serial collection of healthcare environmental dust and water biofilms and define the most common healthcare environmental sources harboring NTM biofilms.

**Trial registration:**

ClinicalTrials.gov NCT05686837.

## Introduction

Among people with cystic fibrosis (pwCF), pulmonary nontuberculous mycobacteria (NTM) is one of the most challenging infections to manage and requires prolonged antibiotic courses which are frequently associated with toxicity and often fail to clear the infection [1, 2]. Positive cultures for NTM were recovered in one in five pwCF over a 5-year period [3]. However, gaps remain in the understanding of cystic fibrosis-specific source(s) of infection, modes of transmission, and exposure risks [4]. Pulmonary NTM infections are thought to be primarily acquired from NTM-rich natural environmental sources including soil, water, water supply systems, and from aerosols generated by flowing water [5–7]. Environmental risk and climatic factors have also been associated with NTM infections among pwCF [8]. NTM have been recovered from municipal water systems and healthcare facilities [9–13]. NTM are found in water and in biofilms formed on pipes, with both being implicated in healthcare-associated outbreaks of infection [11, 14].

Healthcare-associated environmental acquisition and transmission of NTM among pwCF is also a concern among patients and their care teams [15]. We reported healthcare-associated environmental acquisition of *Mycobacterium intracellulare* ssp. *chimaera* among eight pwCF identified by molecular epidemiologic investigation as having respiratory isolates with a genetic species-level match to NTM collected from a hospital water fountain biofilm [11]. The frequency of healthcare-associated transmission of NTM among pwCF is controversial, with studies confirming patient-to-patient transmission [10, 16–20], others finding little to no evidence of transmission [11, 21–23], and some finding transmission only among sibling pairs [24, 25]. While one study concluded that NTM are globally transferred potentially via person-to-person transmission of fomites and aerosols [26], a U.S. multicenter NTM prevalence study concluded that transmission among pwCF was not implicated in the healthcare setting [27]. Prior to 2021, published CF Care Center NTM outbreak investigations were independently designed and implemented, making direct comparisons of findings and conclusions suboptimal. As a first step towards understanding NTM outbreaks among pwCF, the Healthcare-associated links in transmission of NTM (HALT NTM, NCT04024423) study developed an evidence-based epidemiologic investigation toolkit, linked with environmental sampling, watershed analysis, and integrated pan genomic analysis, to standardize investigations of potential healthcare-associated NTM outbreaks within CF Care Centers [28]. The HALT NTM Toolkit tests NTM transmission with whole genome sequencing (WGS) to genetically-match isolates between patients and/or environment with supportive evidence of epidemiologic exposure for potential cross-infection [10, 11, 28].

The parent HALT NTM study was a retrospective parallel multi-site study of pwCF cared for in a single Center who are identified with highly similar respiratory NTM isolates [28]. The Colorado National Resource Centers (CO-NRC) biorepository collected non-standardized, voluntarily submitted respiratory isolates from CF Care Centers throughout the U.S. for the purpose of culture, molecular identification, and whole genome sequencing (WGS). Using phylogenomic analysis of samples banked from 2015–2020, the CO-NRC identified clusters of NTM isolates, defined as highly similar strains at the genomic level, cultured from two or more pwCF who were cared for at the same Care Center [29, 30]. However, NTM isolates were voluntarily submitted in a non-systematic fashion and did not include all isolates from all patients who cultured positive for NTM at their site. Due to this lack of standardization of NTM isolate submission and collection, identification of highly-related NTM clusters was likely underestimated and was identified months to years after potential transmission occurred, limiting timely infectious disease control and inhibiting implementation of potential mitigation strategies.

Herein we describe the Prospective healthcare-associated links in transmission of NTM among people with CF (pHALT NTM, NCT05686837) study protocol, which will systematically screen all respiratory samples collected over a four-year period for NTM in participating CF Care Centers. The scientific premise that forms the basis for the proposed research is that with prospective, longitudinal respiratory and environmental NTM sampling, the timeliness and accuracy of outbreak detection as well as real-time detection of the origins and prevalence of healthcare-associated patient-to-patient transmission and/or acquisition of NTM among pwCF can be determined and appropriate mitigation implemented. This is the first study to prospectively standardize serial respiratory NTM isolate collection from all pwCF receiving care at a participating Care Center combined with serial collection of healthcare environment dust and water biofilms to assess endemic environmental NTM exposures in the healthcare system as well as characterizing shared home of residence watersheds to identify healthcare-associated NTM outbreaks among pwCF in real-time (Table 1).

**Table 1:** Study objectives.

**Primary Objectives**	1. Epidemiologic investigation: a) Assess the origins and prevalence of healthcare associated patient-to-patient transmission and/or acquisition of NTM. b) Characterize the source(s) of patient-to-patient transmission and/or acquisition of NTM within an individual CF healthcare setting.
	2. Environmental investigation [28]: Determine if NTM strains identified in clusters are related to strains isolated from dust and/or water biofilm sources in the CF healthcare setting.
	3. Home of residence watershed mapping: Determine if PwCF infected with clustered NTM isolates live in the same home of residence watershed.
**Secondary Objectives**	1. Epidemiologic investigation: a) Assess degree of proximity of subjects in time and/or space. b) Compare the relationship between similarity of strains and degree of proximity of subjects. c) Model the relationship between the degree of similarity and strains with the proximity and subject characteristic variables. d) Compare the prevalence of respiratory CF NTM species/subspecies by geographic region
	2. Environmental investigation: a) Characterize human disease causing NTM isolates in healthcare dust and water biofilms. b) Describe most common biofilm sources within the healthcare setting harboring NTM isolates that cause human disease. c) Compare the prevalence of healthcare-associated dust and water biofilms NTM species/subspecies by geographical region
	3. Home of residence watershed mapping: N/A

## Materials and methods

### General overview

**Fig 1:**
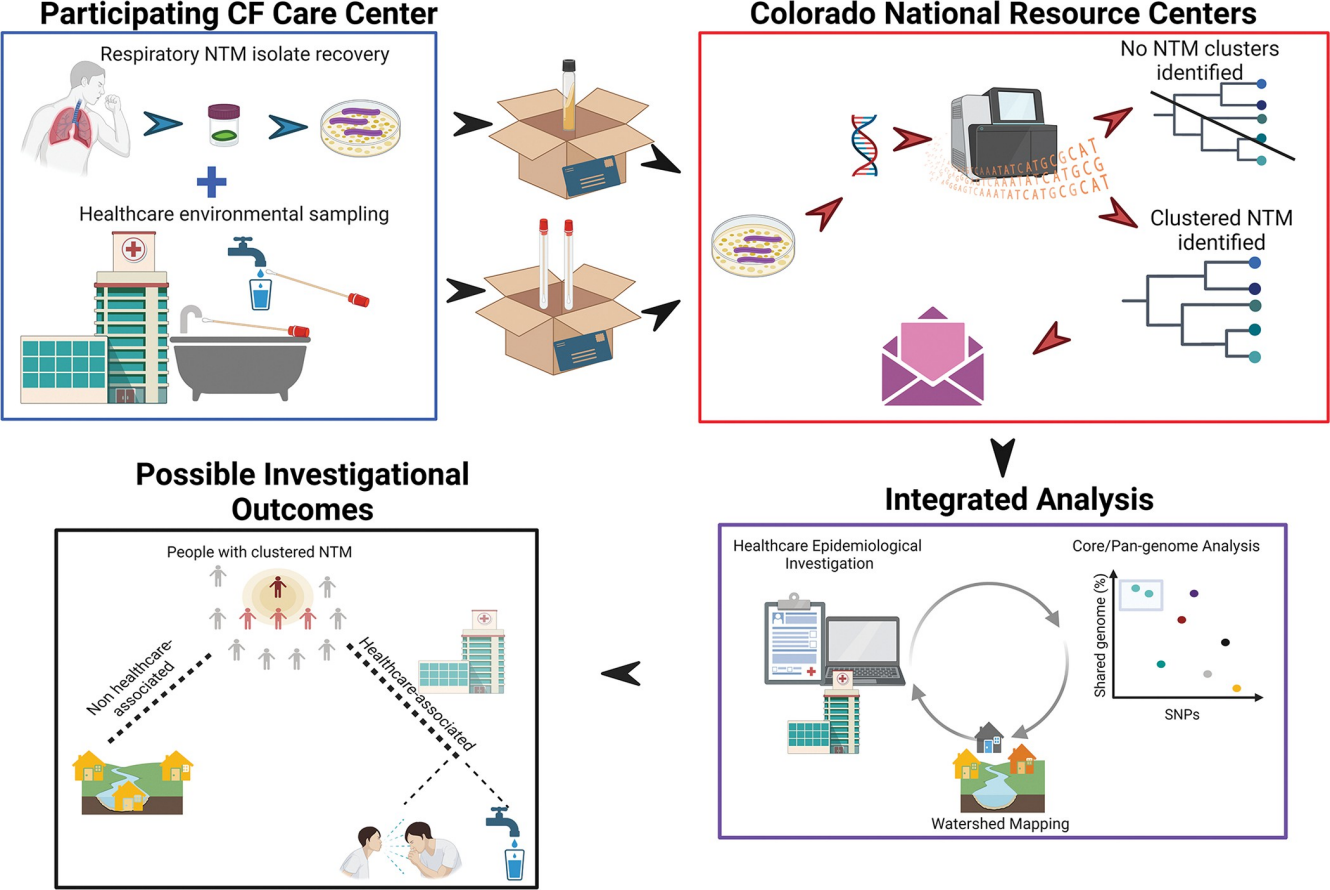
Schematic showing an outline of the prospective healthcare-associated links in transmission of nontuberculous mycobacteria among people with cystic fibrosis (pHALT NTM) study. Respiratory nontuberculous mycobacteria (NTM) isolates from people with cystic fibrosis (CF) and healthcare environmental sampling swabs will be collected from each CF Care Center (blue box) and shipped to National Jewish Health (NJH), where core genome analysis will be performed and NTM clusters will be identified (red box). Integrated analysis is performed in collaboration with the participating CF Care Center and the Colorado National Resource Centers at NJH (purple box). Created with BioRender.com. Reprinted from Biorender.com under a CC BY license, with permission from Mayet Awoke, Customer experience representative, original copyright 2023.

The pHALT NTM study is a prospective, multicenter, nonrandomized study to investigate potential NTM outbreaks in U.S. CF Care Centers. This study will investigate potential episodes of healthcare-associated NTM transmission and/or acquisition, coupled with healthcare environmental sampling and home of residence watershed analysis. An outline of the pHALT NTM study investigation process is outlined in Fig 1. Clusters of NTM isolates are defined as highly similar strains at the genomic level, cultured from two or more pwCF who are cared for at the same Care Center [28, 29, 31]. Highly-related clusters of NTM are defined as ≤20 or ≤30 single-nucleotide polymorphism (SNP) differences at the core genome level, for *Mycobacterium avium* complex (MAC) and *M*. *abscessus* complex, respectively.

National Jewish Health Institutional Review Board (IRB) initially approved the HALT NTM study (#HS-3175-528) on October 9, 2018 and renewed approval on October 4, 2019. Biomedical Research Alliance of New York (BRANY) IRB approved this protocol (#HS-3175-528) on July 1, 2020 and renewed approval on Aug 17, 2021. BRANY approved modifications of HALT NTM to pHALT NTM (#HS-3175-528) Protocol Version 1.2 on Jan 5, 2023. Patient consent is not required for this study.

### Study design

The pHALT NTM study will systematically collect respiratory NTM cultures from all pwCF, as part of routine care, over a 3-year period in CF Care Centers. NTM positive respiratory cultures will be identified by each participating Center by their respective local laboratory. Subject enrollment is unlimited and based on the number of pwCF identified as having an NTM infection identified as having membership in a highly related cluster among individuals receiving care at a participating CF Care Center. The inclusion and exclusion criteria are shown in Table 2.

**Table 2:** Inclusion and exclusion criteria.

Inclusion Criteria	Exclusion Criteria
1. Diagnosis of CF consistent with the 2017 CFF guidelines [32].	1. No formal diagnosis of CF.
2. Male or female participant of any age who has a history of NTM or a first positive NTM culture collected as part of routine clinical care from expectorated sputum, induced sputum and/or bronchoalveolar lavage.	2. A pwCF not followed at a participating CF Care Center.

NTM positive isolates will be sent to the CO-NRC and undergo DNA extraction, WGS, and SNP analysis as previously described [29, 33]. If multiple NTM species or subspecies are recovered from a single respiratory sample from a pwCF, all unique isolates of NTM species or subspecies will be analyzed. The CO-NRC will identify highly similar isolates within Centers by analyzing WGS on all NTM respiratory isolates collected and results will be combined with existing isolate data from each center, if available. The following stepwise process will be utilized: 1) An isolate from each NTM positive respiratory culture from pwCF during the study timeframe will be mailed to the National Jewish Health (NJH) Advanced Diagnostics (ADx) laboratory, where the isolates will be collected by an Honest Broker to de-identify all respiratory NTM isolates received for the study. 2) The de-identified NTM isolates will be cultured, banked, and DNA extracted. 3) The CO-NRC will perform WGS on extracted NTM DNA. Briefly, high quality genomic DNA will be packaged in to sequencing libraries using a tegmentation approach from Illumina, Inc, and MiSeq 600 v 3 kits will be used to generate whole genome data for assembly into 40X coverage genomic assemblies to be used for downstream analysis and comparison. 4) The sequenced DNA will be analyzed at the core genome level to determine relatedness, and respiratory isolates will be considered highly-related and falling into a cluster using a SNP threshold of ≤30 for *M*. *abscessus* subspecies and ≤20 for MAC species as previously described [10]. Phylogenetic trees will be created using longitudinally sequenced isolates from each pwCF and cluster network analysis performed as described [29, 31]. Combined genetic plots will be created, pan genome analyzed, and pairwise accessory genome comparisons made [10, 11].

The HALT NTM Toolkit will analyze date and location data to identify possible acquisition and/or transmission events in the healthcare setting of subjects determined to fall within a cluster based on WGS and phylogenetic analysis [10, 11, 28]. Utilizing the HALT NTM Toolkit, demographic, patient location, microbiological data, and details regarding Center infection prevention and control (IP&C) practices will be collected from the electronic health record (EHR), then assembled and managed using REDCap® electronic data capture tools hosted at NJH [28]. Healthcare data will be used to identify opportunities for transmission events between pwCF infected by clustered NTM isolates based on WGS and phylogenetic analysis. Timeline overlap analysis will be performed [10, 11]. In all participating sites, dust and water biofilm healthcare environmental samples from the clinic, hospital, and research settings will be internally collected at the beginning of the study and every six months over a 2-year collection period. Environmental samples will be mailed to NJH for environmental culture for NTM. If NTM is recovered, isolates will be banked, DNA extracted, WGS performed, and phylogenetic analysis completed [10, 11]. If multiple NTM species or subspecies are recovered from a single environmental biofilm sample, all unique isolates of NTM species or subspecies will be analyzed. Sample analysis will determine: 1) relatedness of environmental NTM over time between the parent HALT NTM study and the pHALT NTM study, 2) relatedness over time with longitudinal sampling during the pHALT NTM study period, and 3) relatedness of respiratory and environmental NTM collected prior to the NTM outbreak investigation. Environmental NTM will be recovered, genomic DNA will be extracted, and isolates will be identified to the species or subspecies level [10, 11]. To assess pwCF common source water exposure in the home environment, patient addresses will be extracted and mapped within watershed boundaries [10, 11]. The following detailed approach will be utilized. Individual sites will employ the HALT NTM Toolkit to perform a confidential, internal, standardized investigation to gather date and location data from the EHR for all pwCF identified by the CO-NRC as infected by clustered NTM isolates. These healthcare data will be used to identify opportunities for transmission events between pwCF infected by clustered NTM isolates based on WGS and phylogenetic analysis. Timeline overlap analysis will be performed [10, 11]. Healthcare environmental samples will be internally collected by each site and mailed to NJH for environmental culture for NTM. If NTM is recovered, isolates will be banked, DNA extracted, WGS performed, and phylogenetic analysis completed [10, 11]. All environmental isolates will be analyzed at the core and accessory genome level to determine relatedness to each other and to respiratory isolates. To assess if common NTM acquisition may have occurred outside the healthcare system and could be attributed to common source water exposure in the home environment, home of residence addresses will be analyzed based on watershed distribution [10, 11].

### Sample size

All U.S. CF Care Centers are eligible for enrollment. The sample size of each cluster, and the number of clusters analyzed, depends on the number of pwCF attending the Center, the number with NTM infection, and the number of available respiratory NTM isolates provided for WGS analysis, and the number of pwCF with NTM infections identified in clusters within each Center. If in fact an outbreak event occurs within a participating Center, it is likely that the number of pwCF having shared infections would be larger. Therefore, sample size will vary within each Center.

### Measures

The following baseline demographic and clinical characteristics will be reported for each subject: age, sex, race, ethnicity, address, zip code, NTM species/subspecies, social contacts with other pwCF, and subject’s CF Care Center(s).

### Epidemiologic investigation

The HALT NTM Toolkit will be used to assess if opportunities for healthcare-associated NTM transmission between patients identified with clustered NTM infections occurred between two or more pwCF within the CF healthcare setting (clinic, hospital, research facility, and common access areas) [28]. An “overlap” is defined as a healthcare setting where two or more pwCF identified in the cluster received care in the same setting for any period of time within 24 hours, as previously described [28]. A point instance of overlap is defined as a specific physical space within the healthcare setting where two or more pwCF identified with clustered NTM infections received care [28].

To evaluate all potential opportunities for healthcare-associated NTM transmission between patients, subjects’ outpatient and inpatient care encounters along with NTM culture data will be plotted in a timeline overlap analysis as described in the parent study [28]. As previously described, subject 1 will be identified as the subject with the longest known isolation of NTM, Subject 2 will be identified as the subject with the second longest isolation of NTM, and so forth. The start date for the potential NTM exposure window will be 2 years prior to study enrollment as was the standard in the parent HALT NTM study [10, 11, 28]. The clinical significance of a first positive CF NTM culture among pwCF that develop NTM disease is demonstrated in lower baseline forced expiratory volume in 1 second (FEV_1_) at the time of first positive culture as well as an increased rate of decline in FEV_1_ in the year preceding the first positive culture [34]. With this understanding, it is reasonable to infer that NTM acquisition may occur up to two years prior to collection of the first positive culture. The end date for the exposure window is the date of sample collection for the highly-related strain that was isolated from Subject 2. Using this approach, the directionality of possible transmission from Subject 1 to Subject 2 can be established, regardless of how many subjects are identified in the cluster. Utilizing the HALT NTM Toolkit, demographic, patient location, microbiological data, and details regarding Center IP&C practices will be collected from the EHR, then assembled and managed using REDCap® electronic data captured tools hosted at NJH [10, 11]. Specific details will include location where phlebotomy, spirometry, imaging, consults, and other healthcare-associated interactions occur.

As described in the parent study [28], the HALT NTM toolkit will be completed by a local CF Center team member familiar with local CF care practices, protocols, terminology, and chart review. We strongly encourage involvement of a CF Research Coordinator and the local IP&C team. Data, including protected health information (PHI), entered by a Center will only be visible to that Center and study consultants at NJH. PHI will be de-identified for analysis.

### Environmental sampling

Similar to the parent study [28], this study will also collect and examine dust and water biofilms from healthcare environmental sources including but not limited to vents, sink faucets, showerheads, shower hoses, ice machines, drinking fountains, patient-utilized coffee machines, and decorative water features in the healthcare environment where pwCF receive care. The number of environmental samples collected depends on the number of dust and water biofilm sources identified. In general, one water biofilm sample will be obtained from each water source and one dust sample from each vent from all healthcare locations identified as potential healthcare sources of NTM acquisition and/or transmission. In a typical patient care room, one sample will be obtained from each sink faucet, shower head and hose, bath faucet, and vent dust. In healthcare setting common spaces, one sample will be obtained from each water source including water dispensers, ice machines, drinking fountains, coffee machines, and decorative water features as well as vent dust. Environmental sampling will be performed every 6 months during the study period. Environmental healthcare-associated dust and water biofilms will be selectively cultured for NTM using standard microbiological approaches [35]. Genomic DNA will be extracted from bacterial pellets [33] and isolates recovered will be identified to the species or subspecies level through amplification and sequencing of the RNA polymerase beta subunit (*rpo*β) gene [7, 35, 36]. Human disease causing NTM will be further analyzed by WGS.

### Outcomes

#### Primary outcome

The primary endpoint of epidemiologic investigation is the identification of shared healthcare-associated source(s) of NTM environmental acquisition or patient-to-patient transmission between pwCF receiving care in a Care Center as in the parent study [28].

The primary endpoint of environmental sampling is identification of healthcare environmental dust and water biofilm NTM isolates that are highly related to isolates recovered from pwCF.

The primary endpoint of home residence watershed mapping is identification of common watersheds among pwCF infected with a clustered NTM isolate.

#### Secondary outcome

Secondary epidemiologic investigation endpoints include: 1) Defining incidence and prevalence of CF NTM species and subspecies by geographical region. 2) Performing between Center comparisons of genetic similarity and understanding patterns of potential transmission and/or acquisition. 3) Banking of isolates for *ex vivo* analysis.

Secondary environmental sampling endpoint includes: Defining incidence and prevalence of healthcare-associated dust and water biofilm NTM species and subspecies by geographical region.

## Quality assurance and monitoring

### Quality assurance

After data have been entered into the study database, data validation checks will be applied on a regular basis including data analysis by the lead biostatistician who will monitor for data outliers and perform data validation. If data entry errors are encountered, the study database will be updated in accordance with the resolved queries. All changes to the study database will be documented in a digital audit trail.

### Monitoring

As previously reported [28], the study Principal Investigator (PI) and the CF Foundation are authorized to monitor the study. By signing the protocol, the Investigator grants permission to the Sponsor (or designee), and appropriate regulatory authorities to conduct on-site monitoring and/or auditing of all appropriate study documentation. Monitoring visits are conducted by representatives of the Sponsor according to the U.S. CFR 21 Part 312 and ICH Guidelines for Good Clinical Practice (GCP) (E6) to ensure investigator compliance to 21 CFR Parts 50, 56 and 312 and to GCP.

The Investigator must make study data accessible to the monitor, other authorized representatives of the Sponsor (or designee), Institutional Review Board/Independent Ethics Committee, and Regulatory Agency (e.g., FDA) inspectors upon request. Hard copy files will not be created. All study information is maintained in REDCap® for a period of five years after database lock. There may be other circumstances for which the Sponsor is required to maintain study records and, therefore, the Sponsor will be contacted prior to removing study records for any reason.

## Data management and data analysis

### Data management

Study personnel at each participating site will enter data from the EHR and CF Foundation Patient Registry corresponding to a participant’s visit into the protocol-specific electronic REDCap® database when the information is available. Participants will be de-identified in the study database to be collected by the Sponsor (or designee), but will be identified by a site number, participant number and initials. For all REDCap® data entry, the time and date stamp will track individuals entering or updating data via an electronic audit trail. The Investigator will be responsible for all information collected on participants enrolled in this study. All data collected during the course of this study will be reviewed and verified for completeness and accuracy by the Investigator. At the completion of the study, a copy of the site-specific REDCap® data will be provided to the participating site.

REDCap® is a secure, HIPAA compliant, web-based application designed to support data capture for research studies. REDCap® is maintained by the REDCap® Consortium comprising over 3,500 institutional partners including NJH, and is administrated locally by the NJH Research Informatics Services that is used as a central location for data processing and management. Access to the database requires user authentication with password. All data are stored on a secure server, and backups are encrypted.

## General analysis plan

Analytical methods will primarily involve informatics techniques. Methods involving statistical inference will be limited as the primary scope of the study is to determine ordinal levels of likelihood of transmission of NTM from patient-to-patient and environment-to-patient, for the observed patients. At this point, we do not plan to make inference for larger populations, except where noted below.

Tests of significance will be conducted to help determine whether observed patterns are likely to have occurred under the assumption of a particular mode of transmission (e.g., patient-to-patient transmission, environmental acquisition); such tests will developed using permutation methods. The number of clusters (parent HALT NTM study vs pHALT NTM study) will be compared using a 2-sample Poisson rate test or analogous permutation test, adjusting counts to rates based on length of time periods used for the pre- and post-periods. The same procedure will be used to compare the average number of subjects per cluster between pre- and post-periods, as well as to compare number of subjects contributing to a particular cluster during pre- and post-periods (adjusting for period lengths).

A timeline for completion of data collection and analysis for a participating Center is shown in Table 3. Upon completion of multi-site enrollment, the aggregate results of epidemiologic investigation and environmental sampling from individual CF Care Centers will undergo meta-analysis as seen in Fig 1.

**Table 3:** Timeline of data collection and analysis.

	Year 1		Year 2		Year 3	
Start-up activities						
IRB approval						
Screen and enroll subjects						
Respiratory isolate collection						
Environmental sampling						
Sample analysis						
Data integration						
Manuscript(s) preparation						

### Primary analysis

The primary endpoints are: 1) Identification of a shared healthcare-associated source(s) between patients in a Care Center with identical NTM isolates. 2) Identification of clinically-relevant NTM isolates and prevalent environmental genotypes with in healthcare setting water biofilms. Every available CF NTM isolate will be analyzed and included along with the prospective study, thus building on all available NTM isolates. Analysis of the individual center data will be completed using both qualitative and quantitative approaches. We will identify a set of variables used to assess degree of proximity of subjects in time and/or space. The first analysis will involve quantifying exposure windows for patients within clusters at a site based on hospital or clinic visits, and use of graphical techniques to help determine plausible transmission settings and transmission windows based on date of conversion and dates of positive culture. Graphical techniques will first be employed to help determine likelihood of patient-to-patient transmission between all pairs of subjects within each cluster. Separate graphs will also be constructed to better understand evidence for potential point-source environmental transmission, as well as potential watershed transmission. Among subjects who have overlapping clinic visits, the degree of physical closeness will be determined and qualitative measures of proximity will be assessed as described in the parent study [28].

### Analysis of secondary outcomes

The secondary endpoints are: 1) Adherence to CF IP&C guidelines. 2) Characterization of points of patient overlap. 3) Incidence and prevalence of NTM species/subspecies within healthcare dust and water biofilms. 4) Determine if biofilm isolates are highly related to isolates recovered from patients. As described in the parent study [28], the incidence and prevalence of NTM species and subspecies within healthcare dust and water biofilms will be characterized and comparisons of environmental NTM biofilm isolates and highly-related patient isolates will be provided.

### Interim analyses

Interim data analyses review will occur semi-annually.

## Discussion

The parent HALT NTM Toolkit facilitates a structured process by which individual CF Care Centers will conduct a standardized, independent, and confidential NTM epidemiologic outbreak investigation of patients identified by the CO-NRC as having clustered NTM infections [28]. The pHALT NTM prospective study design will meet the urgent and significant need to promptly identify, investigate, and mitigate suspected healthcare-associated NTM outbreaks among pwCF. The long-term goals are rapid, real-time detection and mitigation of healthcare-associated NTM outbreaks to reduce NTM risk, inform CF IP&C guidelines, and assure pwCF that care at their Center is safe. The secondary goals are to assess the prevalence and origin of NTM outbreaks from patient-to-patient transmission and/or healthcare-associated environmental acquisition.

Currently there is no clear evidence-based benchmark or threshold of NTM transmission among pwCF. Healthcare-associated patient-to-patient transmission and/or environmental acquisition of NTM may vary among Centers based on variations in the implementation of IP&C measures within an individual Center, as well as nuances in the endemic environmental NTM burden and physical layout and design of the healthcare setting that is unique to each institution. Based on published data [10, 11], we are reassured that healthcare-associated NTM acquisition and transmission are very low in Colorado with only one of 11 clusters demonstrating both evidence of epidemiologically plausible opportunity for healthcare-associated transmission and stringent isolated relatedness criteria (≤10 SNP differences and ≥95% shared accessory genome). In Vermont [11], healthcare-associated patient-to-patient transmission of NTM, specifically *M*. *chimaera* and *M*. *avium*, was not found. However, this investigation supports healthcare-associated environmental *M*. *chimaera* acquisition based on 8 of 9 clustered respiratory isolates having a genetic match with a hospital water fountain isolate. Data from other enrolled Centers are currently undergoing analysis, but it is clear based on preliminary data, epidemiologic findings, and environmental NTM exposure for healthcare-associated transmission and/or acquisition varies among Centers. The pHALT NTM study will be impactful, even if no healthcare-associated transmission and/or acquisition of NTM is observed among pwCF because negative results would validate the current IP&C guidelines. The pHALT NTM study will analyze IP&C measures within each healthcare setting, including use of masks, changes directed toward decreasing patient interactions with healthcare setting water sources, and other recommendations for interactions among pwCF in the healthcare setting.

We recognize that the pHALT NTM study does not include evaluation of free water and soil in the healthcare-associated setting, and also does not include environmental sampling of home and other non-healthcare environmental sources. Although additional healthcare environmental sampling and home environmental sampling are both interesting and may be important, these investigations are beyond the scope of this study. A household-specific study protocol to perform direct dust and water biofilm sampling of homes of pwCF infected with NTM may be an avenue of future research.
